# Home Visiting as an Equitable Intervention for Perinatal Depression: A Scoping Review

**DOI:** 10.3389/fpsyt.2022.826673

**Published:** 2022-03-18

**Authors:** Karen M. Tabb, Brandie Bentley, Maria Pineros Leano, Shannon D. Simonovich, Nichole Nidey, Kate Ross, Wen-hao David Huang, Hsiang Huang

**Affiliations:** ^1^School of Social Work, University of Illinois at Urbana-Champaign, Champaign, IL, United States; ^2^School of Social Work, Boston College, Chestnut Hill, MA, United States; ^3^College of Science and Health, DePaul University, Chicago, IL, United States; ^4^Cincinnati Children's Hospital Medical Center, Division of Biostatistics and Epidemiology, University of Cincinnati College of Medicine, Cincinnati, OH, United States; ^5^Cambridge Health Alliance (CHA), Cambridge, MA, United States

**Keywords:** home visiting, depression, perinatal, postpartum, depressive symptoms

## Abstract

**Objective:**

Maternal mental health disorders are a leading complication of childbirth. While few systems are adequately able to identify and treat depression, people experiencing perinatal depression may benefit from the intervention of home visiting. The intent of home visiting interventions is to alleviate stressors of parenthood for people facing additional risk factors. The aim of this scoping review is to investigate the effect of home visiting on perinatal depression grounded in published studies.

**Methods:**

We conducted a scoping review of the existing literature of studies relevant to perinatal depression and home visiting. We entered keywords in five search databases: MEDLINE, PsycInfo, CINAHL, Social Work Abstracts, and Google Scholar. All relevant literature published between January 1999–December 2019 was reviewed. Duplicates, books, and errata were excluded from the study. As a scoping review, we included all studies published in English describing the inclusion of maternal depression in home visits. We hypothesize birthing people with perinatal depression will benefit from home visiting interventions.

**Results:**

The results from the scoping review and describe the use of home visiting to improve perinatal mental health. An initial 12,652 records were identified in the search. After duplicates were removed, the titles of 2,140 articles were assessed for applicability, however 29 identified for full-text eligibility and were included in this analysis. The majority of the studies included in this review were quantitative (*n* = 23), followed by qualitative (*n* = 3), and mixed methods (*n* = 3). Nearly all studies (*n* = 28) using validated instruments such as the Edinburgh Postnatal Depression Scale to determine depressive symptoms.

**Discussion:**

This review offers preliminary qualitative insights on the efficacy of home visiting for administrating perinatal depression care. Studies show that home visiting programs can provide treatment for perinatal depression and reduce the effects of depression for birthing people. Our review suggests that the efficacy of home visiting programs beginning in the postpartum period are less predictable than prenatal home visiting intervention among various populations, including people experiencing both high-risk and low-risk situations.

## Introduction

Transitioning into the role of parenthood is a complex period underscored by a range of physical, lifestyle, and emotional changes, resulting in increased needs for support for birthing people and their families (1). Due to these immense transitions, emotional distress, psychological distress, anxiety, and depression are commonly experienced among during the perinatal period (from pregnancy up to 12 months postpartum) and are often left untreated. The prevalence of clinically significant depressive symptoms ranges from 12 to 23.1% (2, 3) in most studies. Depressive symptoms, independent of maternal stress, can occur at any time during the perinatal period. Maternal distress is associated with adverse maternal, child, and family outcomes including maternal substance abuse, a heightened risk of child maltreatment, and increased challenges for parents (2–4). Social support is one of several protective factors for maternal distress and depression (5). It is possible that social support can mitigate the impacts of untreated mental health of mothers during the perinatal period through early detection and intervention efforts including depression screening. In parallel, addressing the aforementioned challenges faced by parents through supportive programming, such as home visiting or case management, can promote the well-being of the entire family by potentially helping to reduce perinatal depression and creating long-lasting positive outcomes for both parents and children.

Home visiting programs are a globally implemented approach aimed at increasing positive health outcomes through providing parents with mental health screenings, psychoeducation, case management, and social support within each parent's home environment (6, 7). Home visitors may voluntarily engage families at intentional time points throughout the perinatal period, including pregnancy, birth, and postpartum, with routine visits occurring on a weekly to monthly basis. The exact structure of home visiting program models varies, however there are recognized programs such as Nurse-Family Partnership, Healthy Families, and Parents as Teachers. These models utilize standard assessment, education, and screening tools to inform Home Visitors' tailored responses to families' needs. Home visiting programs typically engage nurses, social workers, paraprofessionals, or trained volunteers in the delivery of services (6, 8). Home visiting is a practical approach to ensure the health and well-being of perinatal women and infants. Although prior studies have demonstrated the positive effects of home visiting on child development and healthy parental practices, Perry et al. (9) assert mothers experiencing depressive symptoms may have limited engagement in home-based services, prompting an increased need for home visiting programs to specifically address maternal depression as an aspect of the intervention (9). While there are several models to address perinatal depression, within home visiting there still remain uncertainties on the effectiveness of this approach.

Home visiting programs are an avenue to address maternal mental health inequality during the perinatal period. Home visiting programs often aim to achieve maternal health equity for populations at risk for poor outcomes. Birthing people with increased risk factors for postpartum depression including elevated depressive symptoms during pregnancy, personal or family history of depression, and low levels of social support are often enrolled into home visiting programs, yet there is limited research examining depression within the context of home visitation (8, 9). The aim of this scoping review is to investigate the existing literature of home visiting and its effects on perinatal depression, in an attempt to explore the relationship between these two factors grounded in published studies. Findings from this study may inform the applicability of home visiting as a supplementary approach to increase the positive mental health outcomes of women and infants enrolled in home visiting programs. Specifically, this scoping review addresses the question, “*How do home visiting programs address and/or treat the adverse symptoms of perinatal (antenatal or postpartum) depression (PPD)?”*

## Methods

In order to determine the capacity of home visiting as a sound intervention for PPD, a scoping review was performed by synthesizing data within a relevant timeframe. Studies were identified through a systematic search of relevant literature in five search databases, published between January 1, 1999 and December 31, 2019, a twenty-year period of reference. No review protocol for this study exists.

### Identifying Relevant Studies

We searched MEDLINE, PsycInfo, CINAHL, Social Work Abstracts, and Google Scholar to gather literature. Keywords searched include 51 combinations of the terms “*perinatal*”, “*pregnancy*”, “*postpartum*”, “*antenatal*”, “*maternal*”, “*depression*”, “*depressive symptoms*”, “*depressive mood*”, “*mood*”, “*mental health*”, “*home visiting*”, and “*home visitation*” thus constructing a comprehensive search which reflects the prevalence of our topic. Our findings excluded duplicate studies, books, errata, and/or studies with insufficient relevance to the research question. Our findings included studies published in English language, that utilized a home visiting sample and included perinatal depression in any capacity. Non-English language papers were not included. The date range of this study includes data published from 1999 to 2019. Authors, KT, BB, and MPL independently charted the data from each eligible article and met weekly to continuously update the data-charting form in an iterative process.

### Literature Selection

**Figure 1:**
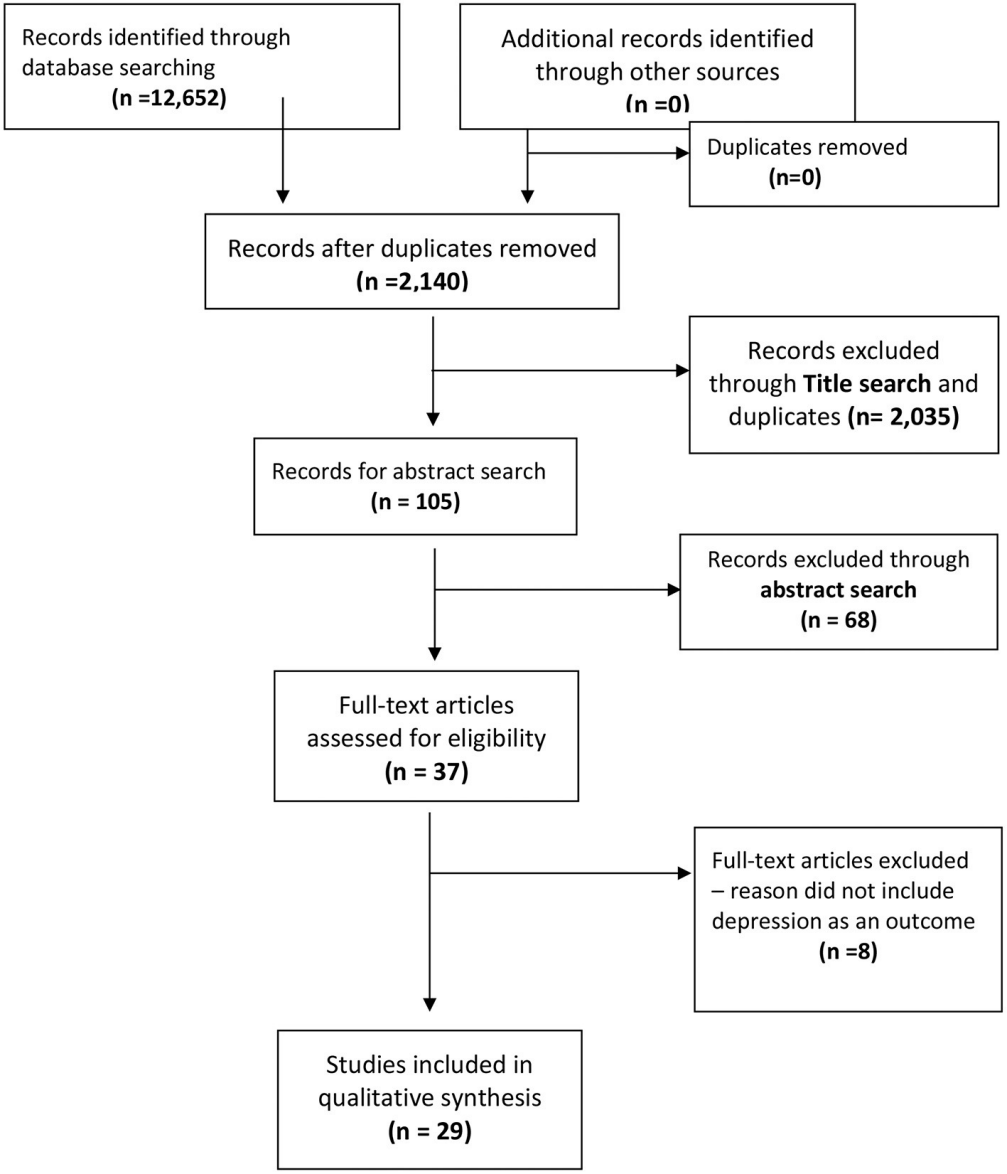
Flow diagram of scoping review search and inclusion.

Initially, 12,652 records were identified. After duplicate records were discarded, 2,140 articles remained. Following title search, 37 articles remained for abstract review. Abstracts were reviewed by authors KT, BB and KR and assessed for inclusion eligibility. Articles that did not include depression as an outcome within the context of home visiting were excluded. A total of 29 studies were included in this scoping review (see Figure 1).

## Results

The results are organized and presented by the timing of the intervention; perinatal or postpartum in Supplementary Table 1, which also summarizes the country, study design, sample population, type, duration and frequency of home visits, type of education provided, assessment of depression, and lastly study outcomes and effect sizes. The majority of the studies included in this review were conducted in the United States (*n* = 16), followed by Australia (*n* = 4). The majority of the studies included in this review were quantitative (*n* = 23). An additional three studies were qualitative, and three were mixed methods. The studies included in this review used different tools to assess depression ranging from qualitative interviews to diagnostic interviews. However, most studies (*n* = 28) included in this review assessed depression using questionnaires. The questionnaire that was most often used in the studies included in this review to assess depressive symptomology was the Edinburgh Postnatal Depression Screening (EPDS, *n* = 16).

### Interventions Initiated During the Perinatal Period

In this scoping review of 29 studies, only 12 studies were conducted during the perinatal period, meaning the study started during pregnancy and extended into the postpartum period. These findings of these 12 studies during the perinatal period describe the long-lasting effects of home visiting programs on improving PPD for a variety of high-risk low-income populations. Most importantly, the majority of studies that including both pregnancy and postpartum periods presented the element of time in their analysis. Description of the key study findings and their relevance to the effectiveness of home visiting to address perinatal depression from pregnancy through postpartum is provided as follows:

Ammerman et al. (8) analyzed data from 806 women who were enrolled in Every Child Succeeds (ECS). Women in this program were high-risk mothers who were enrolled in the program beginning as early as 20 weeks gestation. The majority of providers in the intervention were social workers or had similar professions. The home visiting interventions were delivered over a 2.5–3-year period, with increased contact at the beginning of the intervention. Depressive symptomology was assessed using the Beck Depression Inventory-II (BDI-II) at enrollment and again 9 months after the first visit. At enrollment, mean depressive scores were 12.29, and at 9 months they had dropped to 10.12 (*p* < 0.001). Further analysis demonstrated that 56.5% of mothers who had elevated depressive symptoms at enrollment were no longer depressed at the 9-month assessment (8).

Brock et al. (10) reported findings from a randomized controlled trial that was implemented spanning from the perinatal period from pregnancy through 24 months postpartum. All participants enrolled in the study had elevated depressive symptoms. Fifty-four women were randomized into immediate (*n* = 36) and delayed listening (*n* = 18) visits and completed a 16-week assessment. The researchers used multiple measures to assess depressive symptoms including the Inventory of Depression and Anxiety Symptoms- General Depression, the EPDS, and the Hamilton Rating Scale for Depression to assess depression severity. The results of this intervention demonstrated that both groups, the immediate and the delayed, experienced significant reduction in depressive symptoms. Furthermore, the improvement in depressive symptoms was maintained over time (10).

Dugravier et al. (11) reported the results of a randomized controlled trial conducted during the perinatal period with 440 first-time mothers who were recruited during their 7th month of pregnancy. The participants were high-risk mothers with at least one psychosocial risk factor (e.g., low-income). The intervention consisted of 14 home visits that were delivered by trained psychologists between the 7th month of pregnancy and 3 months postpartum. Participants were randomized into the intervention or the usual care group. Depressive symptoms were assessed using the EPDS at baseline and again at 3 months postpartum, the end of the intervention. The results demonstrated that in the overall sample there was not a statistically significant difference in depressive symptoms between the intervention and the control group. However, *post-hoc* analysis demonstrated that the intervention was more effective for women with certain psychosocial characteristics, including among those planning to raise their children without a father, those whom had low depressive symptoms at baseline, and among women who had more than 9 years of education (11).

Similarly, Hadley et al. (12) reported the findings of a home visiting intervention with 513 participants who enrolled during the prenatal period. Women who participated in this study were mostly high-risk with the majority of participants unmarried and low-income. The intervention duration was 9 months, during which participants received at least three visits and monthly phone calls, all delivered by community health workers. Depressive symptoms were assessed using the EPDS. The results demonstrated that after participating in the intervention, elevated depressive symptoms were present on 13.31% of women; a reduction from 29.28% at baseline (*p* < 0.001).

One study implemented during the perinatal period investigated the effectiveness of a home visitation program on maternal psychological functioning (13). All participants in the study were eligible for Medicaid; with 198 participants of the home visiting program compared to 221 who did not participate. Depressive symptoms were assessed using the CESD before birth and at 1 year postpartum. The intervention was delivered by trained community health paraprofessionals. The intervention consisted of providing participants with parenting skills and improving psychological well-being. The intervention was delivered from before 28 weeks of gestation through the first year postpartum on a monthly basis. The results showed that, at baseline, those who did not participate in the program had significantly higher depressive symptoms, compared to those who participated in the home visiting program (*p* = 0.007). At 1 year, there were no significant differences in depressive symptoms between the intervention and control groups. However, average depressive scores increased more at 1 year postpartum among the non-participants than they did among the home visiting participants (p = 0.012).

A study by Price et al. (14) investigated the effectiveness of a home visiting program among 25 participants who were assigned to either usual care or the intervention group. All the participants were high-risk; under 100% of the poverty line. The intervention consisted of 12 weeks of home visiting delivered by mental health professionals. Depressive symptoms were assessed at baseline and after 12 weeks using the Patient Health Questionnaire- 9 items (PHQ-9). The results demonstrated that there was a significant reduction in depressive symptoms in the intervention group, but not in the usual care group (14).

In a randomized control study conducted over the perinatal period, Samankasikorn et al. (15) randomized 150 teenage pregnant people into a control and an intervention group. All the participants were high-risk; all were teenagers, and most were unmarried. Community health workers delivered the intervention twice per month during pregnancy and once per month during the postpartum period. Depressive symptoms were assessed at baseline, 3 months, and 12 months postpartum using the EPDS. Overall, the results demonstrated that there was no significant difference between the groups on depressive symptom reduction at 3 months. However, *post-hoc* analysis demonstrated that there was a significant difference between intervention and control groups among Latina participants, with significant reduction in depressive symptoms from baseline to 3 months postpartum (15).

Tandon et al. (16, 17) investigated the efficacy of a cognitive behavioral group therapy intervention in reducing depressive symptoms. A total of 78 women were randomized into an intervention and a control group. All women had elevated depressive symptoms and were at-risk for postpartum depression. The intervention was delivered by licensed social workers or clinical psychologists and consisted of standard home visits plus six 2-hour sessions delivered every week in a group. The intervention group also received booster sessions at 3 and 6-months post-intervention. Depressive symptoms were assessed at baseline, 1-week, 3-months (16), and 6-months (17) using the BDI-II. Major depressive episodes were assessed using the Maternal Mood Screener. At 3-months post intervention, the results demonstrated that women in the intervention group had significantly lower levels of depressive symptoms compared to those in the intervention group (*p* < 0.05) (16). The results from the 6-month post-intervention demonstrated that women who received the intervention still had lower depressive symptoms, compared to the control group (*p* < 0.001).

### Interventions Initiated During the Postpartum Period

In our review, 17 studies were conducted during the postpartum period only. Our review suggests that the efficacy of home visiting programs beginning in the postpartum period are less predictable than perinatal home visiting intervention from pregnancy through postpartum, among various populations, including high- and low-risk participants. Armstrong et al. (18) conducted a randomized controlled trial to determine the effectiveness of weekly home visits. All participants were high-risk; with some form of physically abusive relationship with their partners or children in addition to low income status and/or a history of a mental health disorder (18). The intervention delivered by nurses consisted of weekly visits for the first 6 weeks, then every other week through 3 months, and then monthly visits until the child turned 6 months. The EPDS was used to assess maternal depressive symptoms at baseline and then again at 6 weeks. The results indicated that the EPDS scores from the intervention had improved significantly, compared to the scores from the control group (*p* < 0.05). Moreover, the results demonstrated that the intervention had been more effective for primiparous women than for multiparous women (18).

A study by Ahn and Kim (19) investigated the effectiveness of a home visiting discharge education intervention on postpartum depression among birthing participants who had their children in neonatal intensive care units (19). A total of 35 participants were enrolled into the intervention (*n* = 23) and control (*n* = 12) groups. The intervention consisted of a 1-h home visit provided within one-week of hospital discharge. The intervention was delivered by a registered nurse. Depressive symptoms were assessed at baseline and again after the intervention, using the EPDS. The post-intervention results demonstrated statistically significant reduction in EPDS scores in the intervention group but not in the control group (*p* = 0.001).

A postpartum period study tested an informal home visiting support program using a cluster randomized design (20). All participants were living in socially disadvantaged situations. The intervention was delivered by trained community volunteers. The number of visits varied depending on the needs of the participants. Depressive symptoms were measured using the EPDS at 2 and 12 months. Minor and major depression were assessed using a clinical interview. The results indicated that there were no differences in depressive symptoms among the participants in the intervention group, those in the matched-control group, or the unsupported group at 12 months postpartum (20).

Beeber et al. (21) tested the effectiveness of an intervention with participants who were part of an Early Head Start program. A total of 16 participants were randomized into an intervention and a usual care group. All participants included in the study had elevated depressive symptoms, which were assessed using the CESD. The intervention consisted of 8 face-to-face sessions and a booster of weekly telephone sessions delivered by nurses. Depressive symptoms were assessed at baseline, 8 weeks, and 16 weeks in the treatment and control groups. The results demonstrated that the intervention group had significantly lower scores of depressive symptoms at 8 and 16 weeks (21). Depressive symptoms in the control group did not change across time.

A study by Christie et al. (1) investigated the effectiveness of the frequency of home visits using a randomized controlled trial. All participants were primiparous and considered low-risk. The intervention was delivered by health home visitors and consisted of six home visits delivered between 10 and 14 days to 2 months postpartum. Depressive symptoms were assessed at 2 and 7 months postpartum using the EPDS. The results demonstrated that at 2 months, the intervention group had significantly higher mean depressive symptom scores (p = 0.02), compared to the control group. It was also found that at 7 months there was no significant difference in depressive symptoms between the intervention and control groups.

Cust (22) conducted a pilot study to determine whether peer support would aid in reducing postpartum depressive symptoms. Peers had first-hand experience with a history of mild to moderate postpartum depression but had recovered by the time they delivered the intervention. A total of 30 participants were allocated to the control (*n* = 15) and intervention groups (*n* = 15). The intervention consisted of six visits delivered every week. Depressive symptoms were assessed using the EPDS at baseline, after the intervention (or when the child was 3 months), and when the child was 6 months. The results showed that after the intervention, the EPDS scores of the participants in the intervention group were lower than those in the control group. These results were maintained at 6 months (22).

Flemington et al. (23) used a retrospective design to assess the effectiveness of a nurse home visiting program. All participants were high-risk; they had a history of mental illness, or had a drug or alcohol issue, among others. The intervention was delivered from the time the baby was born through 12 months. Maternal depressive symptoms were assessed at enrollment, when the child was 6 weeks, 12 weeks, and 6 months using the EPDS (23). Overall, the results demonstrated that depressive symptoms increased over time.

Another study conducted by Horowitz et al. (24) tested the effectiveness of a behavioral coaching intervention, aimed to improve the mother-child relationship, using a randomized controlled trial. A total of 134 women with elevated depressive symptoms were recruited to participate in the study. Elevated depressive symptoms were assessed at 6 weeks, 3, 6, and 9 months after the baby's birth using the EPDS and the Postpartum Depression Screening Scale (PDSS) (24). The intervention was delivered by nurses and consisted of home visits at 6 weeks, and at 2, 3, 4, 6, and 9 months postpartum. The results demonstrated that although depressive symptoms went down significantly over time, there were no significant differences between the intervention and the control group.

Letourneau et al. (25) assessed the effectiveness of a home-based peer support program among participants who had elevated postpartum depressive symptoms. A total of 33 participants were assigned to the control group and 27 to the intervention group. The intervention consisted of peer support provided over 12 weeks of home visits and contacts over the phone. Peers who delivered the intervention had a history of recovery from postpartum depressive symptoms and received training on providing different types of support. Depressive symptoms were assessed using the EPDS at baseline, 6 weeks and 12 weeks after participants were randomized to the groups (25). The results demonstrated that depressive symptoms were reduced in both the intervention and control groups; however, the intervention effects were not significant.

Milani et al. (26, 27) described the effectiveness of a home visiting program in Iran to address common postpartum issues, including postpartum depression. A total of 92 participants were assigned to the intervention group and 184 to the control group. Depressive symptoms were assessed using the EPDS at 2 months postpartum. The intervention was delivered by midwives and consisted of home visits provided in the first and second weeks postpartum (26, 27). The intervention effects resulted in a lower rate of elevated symptoms among intervention participants (7.6%), compared to those in the control group [19.0%; *p* < 0.05; (26, 27)].

Nugent et al. (28) randomized 106 postpartum women into an intervention (*n* = 57) and control (*n* = 55) group to determine the effectiveness of an intervention to reduce postpartum depressive symptoms. The intervention consisted of routine care at the hospital plus a bedside visit within 2 days postpartum and also a home visit at 1 month postpartum (28). The intervention was delivered by trained psychologists or nurses. Depressive symptoms were assessed using the EPDS at one-month postpartum. The results demonstrated that fewer participants in the intervention group had elevated depressive symptoms (EPDS > 12) compared to the control group. However, the results did not reach statistical significance (p = 0.05, CI = [0.02, 1.11]).

Finally, a study conducted by Paris et al. (29) aimed to identify the effectiveness of an intervention to address maternal mood. A total of 25 mostly primipara participants were enrolled in the study. The intervention consisted of 12 to 16 weekly home visits delivered by trained clinicians from mental health disciplines (29). Maternal depressive symptoms were assessed using the Postpartum Depression Screening Scale (PPDS) and the Beck Stress Inventory (BSI) before and after the intervention. The results demonstrated a statistically significant decrease in total postpartum depressive symptoms (*p* < 0.001). A significant decrease was also observed on the depression sub-scale of the BSI (*p* ≤ 0.05).

Rossiter et al. (30) qualitatively examined the experiences of 111 participants who received home visiting services until their children turned 1 year. Depressive symptoms were measured using the EPDS (30). Only women with elevated depressive symptoms were included in the intervention. Thematic content analysis identified that the most valued aspects of the home visits included the quality of the home visitors (who were nurses); the consistency and continuity of the home visits; the increased parenting confidence; and the increased understanding and bonding that was created between the parent and the infant. Overall, the participants were satisfied with their participation in the home visiting program.

There were a limited number of studies with qualitative data to report. Cust (22) conducted in-depth interviews with participants from their study's intervention and control groups and found that having the support of a peer made the participants feel understood. Horowitz et al. (24) also assessed qualitative information from their participants and found that participants enjoyed the support provided by the home visits. The participants described that they felt validated and became more aware of their emotions and how these could affect their children.

## Discussion

This scoping review describes the summative results of 29 home visiting intervention studies designed to address depression during the perinatal period. This scoping review revealed clear discrepancies between the timing of initiating home visiting and intervention effectiveness for people experiencing depressive symptoms during the perinatal period. Synthesizing the results of these 29 interventions, it is clear that perinatal home visiting programs that begin during pregnancy and continue through the postpartum period report more consistent and favorable outcomes related to reduction in depressive symptoms among childbearing people than home visiting interventions which do not begin until the postpartum period. The discrepancies in the consistency of each intervention included in this scoping review, to a large extent, may be grounded research design factors in which home visiting interventions have been utilized to address perinatal mental health concerns in largely high-risk populations with very limited resources as exemplified in the 12 perinatal studies included in this scoping review. While these studies attempt to achieve health equity and reduce disparities in the burden of perinatal depression among high-risk populations, the research teams often faced additional challenges and barriers that limited the full potential of the intervention. The 17 postpartum studies, however, were derived from both low-risk and high-risk populations. We speculate that the wide range in participants' demographic characteristics might have contributed to the inconsistent predictability of home visiting program outcomes, particularly among the postpartum studies.

Home visiting is one equitable intervention to improve the quality of mental health and access to care for mothers experiencing PPD. Portela et al. (31) note the increasing need to improve interventions aimed to secure positive change in healthcare industries; given most healthcare interventions are complex in nature, require multiple sources of input, and rely upon longitudinal monitoring to understand standards of quality (31). Portela et al. (31) identified four types of improvement intervention studies: effectiveness studies (e.g., quasi-experimental, observational, and systematic review studies), process evaluations, qualitative studies, and economic evaluations. In this scoping review, effectiveness studies represented the majority of data sources, which has somewhat limited our understanding of the holistic efficacy of home visiting programs. Only two qualitative studies presented here aimed to investigate the experiences of mothers receiving home visiting interventions to address depressive symptoms (30, 32) and only three studies (14, 22, 24) employed mixed-methodological approaches. We contend that multiple perspectives of study design would improve the outcome of home visiting interventions necessary to broaden our understanding of how to optimize home visiting programs' design and implementation for various patient populations.

This scoping review has a number of strengths. For example, by searching a 20-year period we found that the number of home visits was not substantially linked to any pattern of improvement in maternal mental health outcomes. This is important for future program decisions to determine the fidelity of the intervention and required number of sessions to improve maternal mental health outcomes. While seven studies derived results using the number of home visits, 17 studies utilized time span, the period between which home visits began and came to an end. Assessing the seven studies which utilized the number of home visits to determine the efficacy of the intervention, it is found that the number of home visits varied greatly with a median number of visits of eight (range 15). In establishing best practice guidelines in the implementation of home visit interventions as a strategy for maternal mental health, the number or duration of visits was not found to be particularly relevant. Despite the strengths, this scoping review is not withstanding limitations. For example, 23 out of 29 studies were based on statistical analyses of quantitative questionnaire where the instruments' reliabilities may vary from population to population. While we recognize the value and feasibility of quantitative measures in clinical settings, sources of quantitative data do pose certain threats to their reliability that researchers must acknowledge. An additional limitation is that the home visiting programs' design and implementations vary greatly from study to study. Such variation has demonstrated the effectiveness of a diverse variety of home visiting programs, with regard to both design and implementation due to clinical feasibility, which nevertheless, could contribute to inconsistent empirical findings.

## Conclusion

Home visiting programs are an opportunity to detect and treat women for depression around the time of giving birth in their own environments. The approach of home visiting can reduce a number of barriers to mental health support, such as travel or childcare, for parents with infants. Home visiting programs beginning during pregnancy and extending into the postpartum period appear to have greater impacts; however, the effectiveness of these programs remains mixed. Overall, home visiting programs have tremendous potential to add structural support and achieve health equity at the system level by prioritizing the utilization of social support. Across many studies in this review, higher levels of supportive reassurance and reliable assistance, offered with added social support, are associated with lower levels of perinatal depression and anxiety. These aspects of social support can bolster parents' self-confidence and sense of community, helping them face the uncertainties of new parenthood. Lastly, the majority of studies included in this review were quantitative designs and thus lack the description of the views and experiences of home visitors and their participants. Future studies are needed to gain participants' perspectives of home visiting and factors for participation in home visiting while experiencing perinatal depression.

## Author Contributions

KT, BB, WHH, and HH designed the search protocol and study questions. KT, BB, and MP independently charted the data from each eligible article and met weekly to continuously update the data-charting form in an iterative process. All authors reviewed the final manuscripts, wrote, and edited the current draft.

## Funding

L60 MD008481 award to KT from the National Institute of Minority Health Disparities.

## Conflict of Interest

The authors declare that the research was conducted in the absence of any commercial or financial relationships that could be construed as a potential conflict of interest.

## Publisher's Note

All claims expressed in this article are solely those of the authors and do not necessarily represent those of their affiliated organizations, or those of the publisher, the editors and the reviewers. Any product that may be evaluated in this article, or claim that may be made by its manufacturer, is not guaranteed or endorsed by the publisher.
