# Supplementary material for: Home Visiting as an Equitable Intervention for Perinatal Depression: A Scoping Review
Source: Front Psychiatry. 2022 Mar 18;13:826673. doi: 10.3389/fpsyt.2022.826673 (PMC8971366; doi:10.3389/fpsyt.2022.826673)
Supplement: Supplementary file 1 [file Table_1.pdf]

Table 1: Research Studies included in Scoping Review of Home Visiting Programs to Treat Perinatal Depression

Author(s), Year	Country	Study design	Sample/ Population			n=	Type of home visitor	Type of education	# of visits	Visit intervals	Visit timing		Assessment of depression	Outcomes and Effect Size
			At-risk	*First-time*	*Depressed*						*Perinatal*	*Post-Partum Only*		
Ahn, 2004 [1]	Korea	Quasi-Experimental Randomized Control Trial	x			35	Unknown	Maternal self-esteem, attachment, postpartum depression and family function in the mothers of NICU infants	1	Single visit within one week of discharge from NICU		x	EPDS	There was a decrease in EPDS scores after the home visiting education while EPDS score was slightly increased after the routine hospital-based discharge education, using the Mann-Whitney U test (Z = 47.500, p= .001).
Armstrong, 1999 [2]	Australia	Randomized Control Trial				181	Nurse	Enhance parent self-esteem and confidence, guidance on child development and health care, and increase awareness of community services	12	Weekly for first 6 weeks postpartum, bi-monthly until 3 months, monthly until 6 months		x	EPDS	Intervention group's postpartum depression screening scores were significantly lower than those in the comparison group (F1,169 = 7.35, P< 0.05) following 6-week participation in home visiting (HV) program.
Barkin, 2019 [3]	USA	Quantitative Pre-Test/Post-Test Design		x	x	402	Volunteer mothers	Maternal self-esteem and confidence	Up to 75	Weekly		x	PHQ-9	Depression scores significantly improved after participation in the program. Cohen's d for the PHQ-9 was 0.86.
Beeber, 2004 [4]	USA	Randomized Control Trial	x		x	16	Psychiatric Mental Health Nurse	Strategies for managing depressive symptoms, improving problematic life issues, increasing access to social support, and parenting effectively while symptomatic	8 + 5 telephone Calls	Weekly		x	CES-D	Substantial drop in depressive symptom severity from baseline to second screen in the intervention group and the reduction was sustained at third screen. Control group had level of depressive symptoms stable across three time points.<br><br>The intervention group differed significantly from the usual care/waiting list group in the change in depressive symptoms over time (Wilks'lambda group-by-time interaction F[2, 13] = 6.395, p = .012).
Christie, 2011 [5]	Ireland	Randomized Control Trial		x		102	Health Visitor	Family assessment, health promotion, support, and therapeutic interventions	6	Weekly from 10-14 days to 8 weeks postpartum		x	EPDS	No EPDS difference between the groups was determined at 7 months. The intra-class coefficient was 0.01.

Cust, 2016 [6]	UK	Mixed Methods			x	30	Peer Support Worker	Unknown	6	Weekly		x	EPDS	Intervention group displayed lower EPDS scores after 12 weeks compared to control group (control group = 12.46, intervention group = 10.33 - a difference of 2.13).<br><br>Qualitative findings suggest mothers valued social support, empathy, and shared experiences with the Peer Support workers.
Cwikel, 2018 [7]	Israel	Demographic Analysis, Quantitative Survey	x	x	x	137	Volunteer mothers	Emotional, informational, and role modeling support	Up to 52	Visits conducted weekly up to one year postpartum		x	EPDS	After one year in the HV program, 79.2% of mothers with previously positive EPDS scores reported they were well and functioning at home and at work.
Flemington, 2015 [8]	Australia	Mixed Methods	x		x	40	Child Health Nurse	Maternal-infant attachment, parental adoption of health promoting behaviors, positive parenting practices, reduce parental stress and improve maternal mood, reduce child abuse, and promote the use of community support	Unknown	12 month duration (24 months for Aboriginal and Torres Islander families)		x	EPDS	Despite strong involvement in the HV program, some mothers' symptoms of depression increased over time.
Horowitz, 2013 [9]	USA	Randomized Control Trial			x	134	Nurse	Relationship-focused behavioral nursing intervention	6	6 weeks, 2, 3, 4, 6, and 9 months postpartum		x	EPDS PDSS	No significant difference between treatment and control groups. However, there was a decrease in depression symptom severity across time.<br><br>Cronbach's alpha for the EPDS for 134 participants at baseline (6 weeks) was .82.<br><br>Cronbach's alpha for the PDSS for 134 participants at baseline (6 weeks) was .94.
Letourneau, 2011 [10]	Canada	Randomized Control Trial			x	60	Peer Support Worker	Peer support and maternal-infant interaction intervention	Unknown	12 weeks		x	EPDS	Maternal–infant interaction teaching by peers not well received by mothers with postpartum depression. Significant differences and medium effect sizes favoring the control group were observed for measures of symptoms of postpartum depression ($F = 5.51$, $P = 0.02$).

														Moreover, when the screening EPDS scores were added to the multivariate model (i.e.using four time points), the relationship favoring the control group was even stronger (F = 5.51, P = 0.02, partial eta-squared = 0.10).
Milani, et al., 2017 (a) [11]	Iran	Randomized Control Trial				276	Midwife Home Visitor	Postpartum home care services	Unkn own	Visits conducted over 60 days		x	EPDS	Postpartum home visits had a positive effect on lowering postpartum depression.<br><br>In terms of postpartum depression, 92.4% of the intervention group reported an EPDS score of <10 (no depression), compared to 81.4% of the control group.<br><br>The difference between the two groups was reported as statistically significant (P < 0.05).
Milani, et al., 2017 (b) [12]	Iran	Comparative Study And Clinical Trial				276	Midwife Home Visitor	Postpartum health care services	Unkn own	Unknown		x	EPDS	Prevalence of postpartum depression was lower in the intervention group. In this study, the postpartum depression rate was 7.6% in the intervention group which had health-care services at home and 19% in the control group on the 60th day after delivery. The result showed that there was a significant difference between these two groups (P < 0.05).<br><br>By adjusting the significant variables in postpartum depression and using logistic regression test, effective variables in the postpartum depression consisted of group variable which was statistically significant at P < 0.1 and odds of depression in the group who had no intervention was 2.1 times more than group with home visiting.
Nugent, 2014 [13]	USA	Randomized Control Trial		x		106	Unknown	Positive mother-infant relationship	Unkn own	Visits conducted two days post-delivery to 1 month post-delivery		x	EPDS	“Newborn Behavioral Observations” (NBO) program associated with lowering the odds of depressive symptomatology by approximately 75%.

														Significantly fewer mothers had elevated depression symptoms in the intervention group (OR=0.20; 95% CI [0.02, 1.11]; p = .05).
Paris, 2011 [14]	USA	Qualitative Pre-Test/ Post-Test Self Report Surveys	x	x		32	Unknown	Psychotherapeutic treatment	16	Weekly over 17 weeks		x	PDSS, BSI, PSI-SF, MSI-SF	"Early Connections" program was associated with improvements on mother's self- report of depression, psychological distress, and maternal perceptions of the parenting experience.<br><br>At posttreatment, most mothers reported significantly lower PDSS scores, with a mean score falling below the cutoff point for clinical levels of PPD. (Pretreatment 116.71 (25.52), posttreatment 66.71 (22.51) t = 5.79)
Price, 2015 [15]	USA	Mixed Methods	x			14	Maternal and Child Health Home Visitor (MIECHV)	Grief and loss, relationship conflict, adapting to parenting, and understanding and coping with depression	Unkn own	Visits conducted over 6 months		x	PHQ-9<br><br>focus groups using open-ended "stem" questio ns and intervie w coding	Quantitative results indicated statistically significant decreases in depressive symptoms were associated with the "Enhanced Engagement" (EE) HV intervention.<br><br>For the outcome variable of depressive symptoms measured on the PHQ-9, the group × time interaction effect demonstrated a decrease in depressive symptoms associated with the EE intervention that approached statistical significance (p = 0.0600).<br><br>Qualitative results highlighted the importance of open conversation, genuine interest, and normalizing of experiences
Rossiter, 2012 [16]	Australia	Qualitative Satisfaction Survey			x	111	Nurse	Enhance parenting strengths, maternal bonding, and connection with infant	Unkn own	Visits conducted over about 12 months		x	EPDS	Most valued elements of the HV program included home visitors' knowledge, compassion, and reassurance, and improved confidence and emotional responsiveness of mothers
Taggart, 2000 [17]	Australia	Qualitative				25	Volunteer	Befriending, listening, assisting with daily activities, and community service connections	Unkn own	Unknown		x	N/A	Mothers highlighted the value offered by HV volunteers and appreciated their friendship and commonalities of motherhood

Author(s), Year	Country	Study design	Sample/ Population			sample size, n=	Type of home visitor	Type of Education	# of visits	Visit intervals	Visit timing		Assessment of depression	Outcomes and Effect Size
			At-risk	*First-time*	*Depr-essed*						*Perinatal*	*Post-Partum Only*		
Ammerman, 2009 [18]	USA	Quantitative Pre-Post Test Measurement Intervention Group Only		x		806	Nurse Social Worker	Nutrition education and substance use reduction, support parents In providing children with a safe, nurturing and stimulating home environment, optimize child health and development, link families to services and promote economic self-sufficiency	Unknown	Visits conducted over 9 months	x		BDI-II	56% of mothers with elevated depressive symptoms recovered (no longer elevated symptoms of depression) by month 9.
Barnes et al., 2009 [19]	UK	Randomized Control Trial	x			250	Volunteer Parents	Parent support in the form of childcare assistance, companionship, and providing parenting advice	Mean of 5.1	Visits conducted over a mean of 5.5 months	x		Structured Clinical Interview, EPDS	No significant differences were found between the three groups at baseline or at 12 months on depression or depressive symptoms.
Brock, R. L., et al., 2017 [20]	USA	Randomized Control Trial	x		x	54	Public Health Nurse	Empathic listening and collaborative problem solving	Unknown	Visits conducted over 8 weeks	x		HRSD, EPDS, Structured Clinical Interview	"Listening visits" associated with significant improvement in mood, mean depression scores decreased across the 8-week study period. HRSD: During Phase II, both groups improved over time, $F(1, 52) = 9.99$, $p = .003$, and improved at a similar rate, $F(1, 52) = 1.25$, $p = .268$.<br><br>EPDS: During Phase II, both groups improved over time, $F(1, 52) = 4.15$, $p = .047$, and improved at a similar rate, $F(1, 52) = 0.14$, $p = .713$.

Dugravier, 2013 [21]	France	Randomized Control Trial		x		440	Nurse	Promote mental health and attachment quality, provide social and emotional support, and address depression	14	6 visits after 7th month of pregnancy; 8 during the first 12 weeks postpartum	x		EPDS	At three months postpartum, mean (SD) EPDS scores were 9.4 (5.4) for the control group and 8.6 (5.4) for the intervention group (p = 0.18). The difference between the mean EPDS scores was 0.85 (95% CI: 0.35; 1.34). The intervention group had significantly lower EPDS scores than controls in certain subgroup.
Hadley, 2014 [22]	USA	Retrospective Design				500	Bilingual Community Health Worker	Supportive case management, health care referrals, information on child development, parenting skills, and public benefits	Minimum of 3; Mean of 6	Visits (unknown interval) supplemented by monthly phone calls.	x		EPDS	Significant reduction in depressive symptomology.<br><br>After receiving services from MOMobile for 6 months on average, only 13.31% of enrolled mothers were still above that cut-off score postpartum.<br><br>This decline was statistically significant [p value < .001).
Lutenbacher, 2018 [23]	USA	Randomized Control Trial	x			188	Peer Mentor	Health education, assist families with navigating systems, making referrals, and direct medical service provision	Less than 10	Monthly home visits from pregnancy through six months of child's age	x		EPDS	Positive and statistically significant (p<0.01) effects were observed on levels of depressive symptoms.<br><br>Compared to the minimal education intervention (MEI) group, women in the Maternal Infant Health Outreach Worker (MIHOW) group demonstrated a statistically significant greater decrease in scores between the baseline and prenatal assessments with the values remaining lower throughout the

														postpartum period (d = 0.57, p < 0.001).
Navaie-Waliser, 2000 [24]	USA	Randomized Control Trial	x			419	MIECHV Home Visitor	Encourage the use of preventive health services, foster healthy behaviors, help improve parenting skills, enhance psychological health	Unknown	Visits conducted from prior to 28 weeks gestation through first year postpartum	x		CES-D	Less depression and higher self- esteem after intensive HV intervention.<br><br>Intervention participants with more intensive home visitor support were significantly less depressed 1 year after delivery, after adjusting for control variables (p = 0.015).
Olds, 2019 [25]	USA	Randomized Clinical Trial	x			618	Nurse	Promote women's prenatal health, improve child's health and development, and enhance parents' health and life course	Mean of 7 during pregnancy; mean of 26 during the first 2 years postpartum	Unknown	x		Beck Depression Inventory	The program had no effects on reports of maternal depression
Samankasikorn, 2016 [26]	USA	Randomized Control Trial	x			150	Community Health Worker	Social support, role modeling, screening, health education, and referrals	Unknown	Visits conducted for about 15 months during pregnancy through 1st year postpartum	x		EPDS	Higher self-esteem and a decrease in depression symptoms were linked to social support. Cronbach's alpha. For EPDS scores, was 0.76 at baseline and 0.83 at 3 months postpartum.
Tandon, 2011 [27]	USA	Randomized Control Trial	x			78	Unknown	Standard home visiting services plus the adapted 6-session version of the Mothers and Babies Course	Unknown	Visits conducted over 6 weeks	x		Beck Depression Inventory	"Standard HV" program associated with decrease in depressive symptoms.<br><br>At the 3-month post-intervention assessment, the researchers found that 84% of intervention participants exhibited improvements in depressive symptoms compared with 41% of control participants.
Tandon, 2014 [28]	USA	Randomized Control Trial	x			61	Unknown	Standard home visiting services plus the adapted 6-session version of	Unknown	Visits conducted over 6 weeks	x		Maternal Mood Screener	"Standard HV" program associated with decrease in depressive symptoms.

								the Mothers and Babies (MB) Course						At 6 months post-intervention, 32.4 % of women receiving usual care were assessed as having a depressive episode compared with 14.6 % of women receiving the MB Course (x^2 = 3.33, P = 0.07, effect size = 0.21).
Tandon, 2018 [29]	USA	Randomized Control Trial	x		x	120	Trained Home visitors	Maternal mood and stress and mother-infant attachment	Unknown	Visits conducted bi-weekly for at least six months	x		Beck Depression Inventory II	"Mothers and Babies" HV intervention participants had decreased depression scores between baseline and 3 months and 6 months postpartum (unstandardized coefficient = -5.99, z = -2.39, P<. 05).
